# Fatty Acid-Rich Extract from *Holothuria atra* for Hyperuricemia via Expressions Modulation of GLUT9a and GLUT9b in Rat Model

**DOI:** 10.3390/molecules28103981

**Published:** 2023-05-09

**Authors:** Ikhsan Ikhsan, Rinaldi Idroes, Azharuddin Azharuddin, Rosnani Nasution, Rika Yusnaini, Muhammad Iqhrammullah

**Affiliations:** 1 Graduate School of Mathematics and Applied Sciences, Universitas Syiah Kuala, Banda Aceh 23111, Indonesia; ikhsanmah@mhs.unsyiah.ac.id (I.I.); rikayus@mhs.unsyiah.ac.id (R.Y.); 2 Department of Surgery, Tgk. Chik Di Tiro General Hospital, Sigli 24116, Indonesia; 3 Department of Chemistry, Faculty of Mathematics and Natural Sciences, Universitas Syiah Kuala, Banda Aceh 23111, Indonesia; rosnani@usk.ac.id; 4 Department of Pharmacy, Faculty of Mathematics and Natural Sciences, Universitas Syiah Kuala, Banda Aceh 23111, Indonesia; 5 Department of Orthopedic and Traumatology, School of Medicine, Universitas Syiah Kuala, Banda Aceh 23111, Indonesia; azharspbo_kspine@yahoo.com; 6 Department of Orthopedic and Traumatology, Dr. Zainoel Abidin Hospital, Banda Aceh 24415, Indonesia; 7 Department of Psychology and Nursing, Faculty of Medicine, Malikussaleh University, Lhokseumawe 24351, Indonesia; 8 Faculty of Public Health, Universitas Muhammadiyah Aceh, Banda Aceh 23245, Indonesia; m.iqhram@oia.unsyiah.ac.id

**Keywords:** arachidonic acid, echinodermata, *Holothuria atra*, oleic acid, serum uric acid

## Abstract

An edible sea cucumber *Holothuria atra* has been hypothesized to have medicinal benefits against hyperuricemia owing to its bioactive compounds, including mono- and poly-unsaturated fatty acids. Herein, we aimed to investigate the fatty acids-rich extract produced from *H. atra* to treat hyperuricemic rats (*Rattus novergicus*). The extraction was carried out using n-hexane solvent and then administered to potassium oxonate-induced hyperuricemic rats, with allopurinol acting as a positive control. The extract (50, 100, 150 mg/kg body weight) and allopurinol (10 mg/kg) were administered QD through an oral route using a nasogastric tube. Serum uric acid, creatinine, aspartate aminotransferase (AST) and alanine aminotransferase (ALT), and blood urea nitrogen of the abdominal aortic blood were investigated. Our results suggested that the extract was rich in polyunsaturated (arachidonic acid) and monounsaturated fatty acids (oleic acid), in which its administration of 150 mg/kg could significantly reduce serum uric acid (*p* < 0.001), AST (*p* = 0.001), and ALT (*p* = 0.0302). The anti-hyperuricemic activity could be associated with the modulation of GLUT9 by the *H. atra* extract. In conclusion, the n-hexane extract from *H. atra* is a potential serum uric acid-lowering agent targeting GLUT9, where further investigations are crucially warranted.

## 1. Introduction

Hyperuricemia, a condition where serum uric acid is retained at over 7 mg/dL, is a noncommunicable disease caused by either genetic or non-genetic factors [1]. As a metabolic disorder, hyperuricemia is a risk factor for gout, kidney disease, diabetes, hypertension, nephrolithiasis, heart disease, and metabolic syndrome [2]. Using a secondary database, a research group concluded that Asian descents are more at risk to develop hyperuricemic conditions [3]. A study on the Indonesian population suggested that age, gender (female), food consumption, stress level, and previous hyperuricemia incidence are predictors for developing hyperuricemic conditions [4,5]. More than 14.4% of the adult population around the world suffered from hyperuricemia [6], where higher prevalence was found in older populations [7,8]. As of today, xanthine oxidase inhibitors (i.e., allopurinol and febuxostat) are prescribed as first-line therapies to reduce the serum uric acid level [9]. Intake of the foregoing xanthine oxidase inhibitors could cause drug hypersensitivity which is manifested in skin rash, looseness, hepatitis, and interstitial nephritis [10,11]. In addition to its production inhibition, serum uric acid level could be reduced by blocking its reabsorption, thereby promoting its excretion. Several uricosuric agents act by modulating URAT1, GLUT9, and organic anion transporter 1 (OAT1) [2]. In particular, GLUT9, a member of glucose transporter family that is encoded by SLC2A9, is responsible for regulating urate reabsorption [12]. In studies observing the mutations in gene SLC2A9, disabling GLUT9 function could lead to the development of type 2 familial renal hypouricemia [13,14]. Therefore, researchers have purposed this protein as a therapeutic target for uricosuric drugs [15]. As an alternative to current pharmaceutical treatment, lower levels of serum uric acid could be achieved by modifying dietary patterns [16]. In hyperuricemic patients, the contents of saturated fatty acid, monounsaturated fatty acid, and polyunsaturated fatty acid were significantly lower as compared to the normal group [17]. The health benefits of consuming mono- and poly-unsaturated fatty acids include the neutralization of plasma pro-inflammatory cytokines [18]. A case-control study of Caucasians with non-alcoholic fatty liver disease revealed a significant association between high serum uric acid clearance and high unsaturated fatty acids intake [19].

*Holothuria atra*, an edible sea cucumber species, contains various bioactive compounds and has been investigated for its bioactivities (including immunostimulatory, hepatoprotection, and cytotoxicity) [20,21,22]. The species is also a well-known source of essential fatty acids (such as omega-3 and -6), playing an important role in its utilization as a functional food [23]. Previously, fatty acids from sea cucumber have been witnessed to ameliorate hyperglycemic conditions by downregulating α-glucosidase [24]. In a previous report, antibiofilm activity against *Planomicrobium* sp. was observed in sea cucumbers—derived fatty acids [25]. The extract from *H. atra* was previously reported to limit the growth of the enteric pathogen *Pseudomonas aeruginosa* [26]. Nonetheless, the fatty acids deriving from *H. atra* have never been investigated for their anti-hyperuricemia therapeutic activity. Therefore, in this present study, we performed an in vivo experiment of fatty acids-rich extract from *H. atra* as anti-hyperuricemic therapy.

## 2. Results

### 2.1. GC–MS Results

Chemical contents in the n-hexane extract from *H. atra* were identified using GC–MS and the results have been presented in Table 1. As much as 44.61% of the extract content was predictively occupied by a polyunsaturated fatty acid—arachidonic acid. Its derivative (eicosanoic acid, methyl ester) was found to have a relative peak area of 1.44% and appeared in the chromatogram after 22.977 min retention time. Other fatty acids with a peak area of around 5–6% included the 2-pentadecyn-1-ol (5.99%); pentadecanoic acid, 14-methyl-, methyl ester (6.62%); heptacosanoic acid, methyl ester (5.38%); and (6*Z*,9*Z*,12*Z*,15*Z*)-methyl octadeca-6,9,12,15-tetraenoate (6.35%). Oleic acid, a monounsaturated fatty acid, appeared twice in the chromatogram after 19.267 and 23.153 min, respectively, with a combined spectral peak area of 5.88%. Taken altogether, the GC–MS results confirmed the rich content of fatty acids in the n-hexane extract from *H. atra*.

### 2.2. Serum Uric Acid

**Figure 1:**
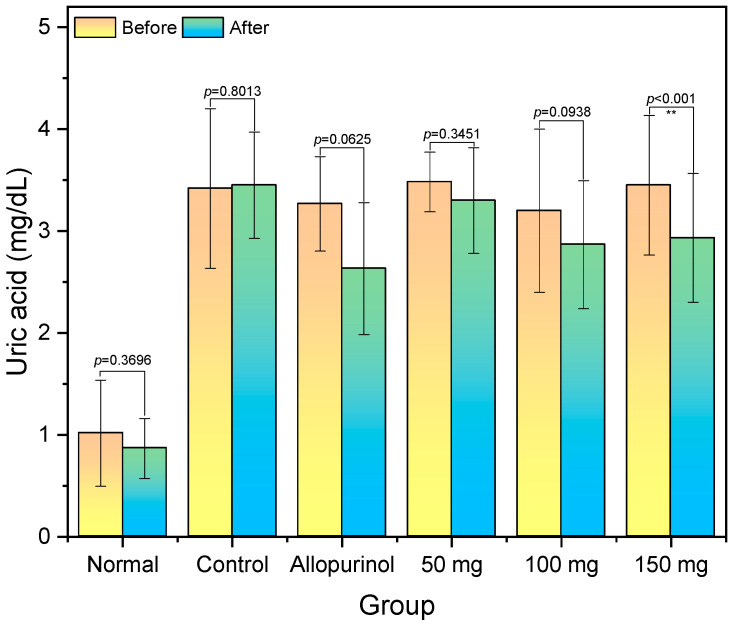
Serum uric acid levels of hyperuricemic rats before and after the treatment. ** Statistically very significant at *p* < 0.01 based on paired *t*-test.

The effect of the n-hexane extract from *H. atra* with dosages of 50–150 mg on serum uric acid in potassium oxonate-induced hyperuricemic rats has been presented (Figure 1). The potassium oxonate injection dramatically elevated the serum uric acid more than three times the baseline. Thereafter, no significant reduction of serum uric acid in control (receiving no treatment), whilst those treated with allopurinol have a reduction with a thin statistical significance (*p* = 0.0625). When treated with *H. atra* extract with concentrations of 50–100 mg, the serum uric acid had a reduction, but was not statistically significant. The statistical significance of serum uric acid reduction was obtained in group 150 mg.

### 2.3. Expression of GLUT9a and GLUT9b

**Figure 2:**
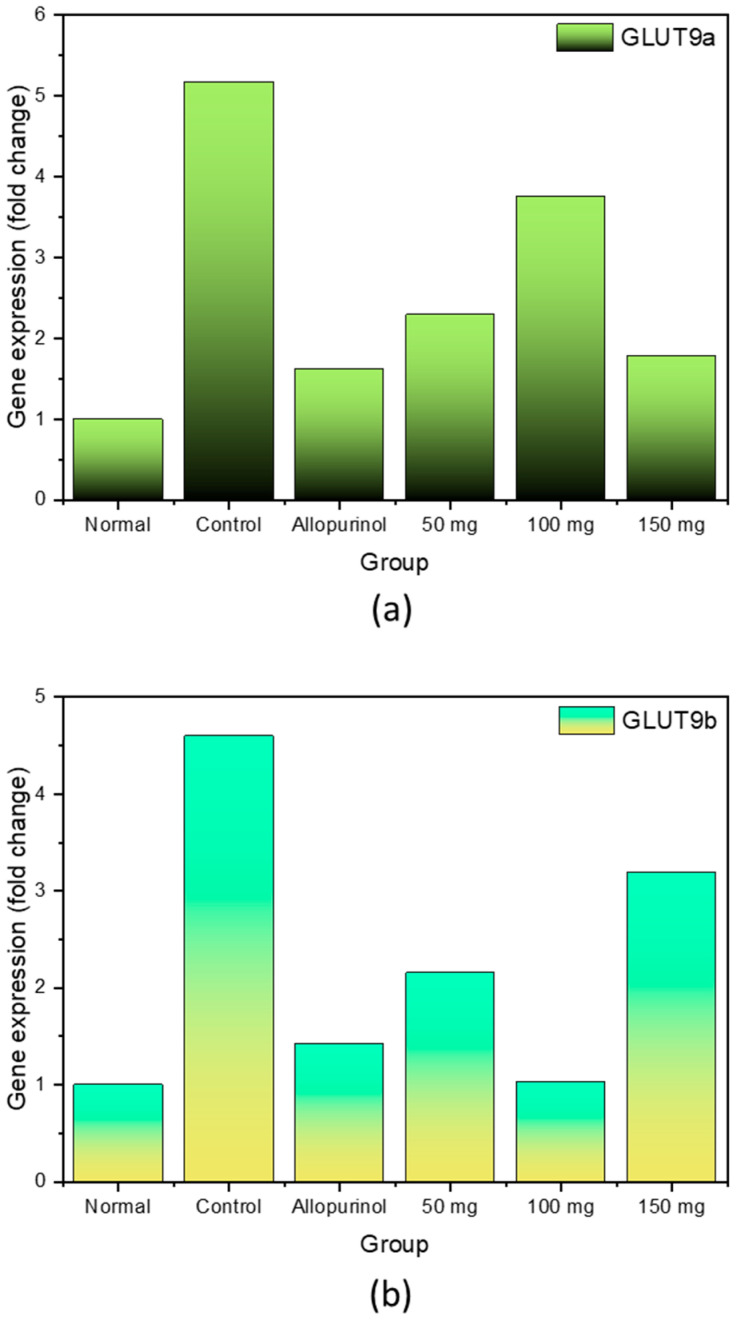
Gene expressions of GLUT9a (**a**) and GLUT9b (**b**) in hyperuricemic rats after the treatment.

Expressions of GLUT9a and GLUT9b in all studied groups have been presented in Figure 2. Expressions of GLUT9a and GLUT9b increased rapidly following the hyperuricemia induction using potassium oxonate. The gene expression of both glucose transporters dropped significantly following the administration of allopurinol. In hyperuricemic rats treated with 50 mg *H. atra* extract, the expression was lower as compared with the control, but still relatively higher when compared to the normal group. Interestingly, when the extract dosage was increased to 100 mg, the expression profiles are different between GLUT9a and GLUT9b, where the former became higher and the latter became lower (compared with group 50 mg). Extract dosage of 150 mg reversed this trend, where GLUT9a expression dropped (to even close to that of group allopurinol) and GLUT9b expression increased (becoming the highest among the extract-treated groups).

### 2.4. Liver Parameters

Serum levels of aspartate aminotransferase (AST) and alanine aminotransferase (ALT) of hyperuricemic rats observed before and after the treatment have been presented in Table 2. Injection of potassium oxonate relatively increased the AST and ALT levels. A significant reduction with *p* = 0.019 was observed in the allopurinol group. Meanwhile, the reduction of AST from 216.0 ± 44.96 to 148.8 ± 20.07 IU/L reached statistical significance at *p* < 0.01 in group 100 mg. A higher significant reduction was observed in group 150 mg (*p* = 0.001). Not only in the case of AST but also the ALT level in group 150 mg also experienced a significant depletion with *p* = 0.0302.

### 2.5. Kidney Parameters

The effect of the extract on blood urea nitrogen (BUN) and serum creatinine levels in the hyperuricemic rat model has been presented (Table 3). Reduced BUN level from 21.80 ± 13.22 to 17.40 ± 5.814 mg/dL with statistical significance was observed in group 50 mg (*p* = 0.022). BUN level was found to be almost significantly lower after the treatment in group 150 mg with a *p*-value of 0.0577. No meaningful change was observed in the case of serum creatinine levels among all investigated groups. All animals survived at the end of the experiment, hence no dropouts.

### 2.6. BSLT Cytotoxicity

Cytotoxicity of the n-hexane extract from *H. atra* was observed in vitro using the brine shrimp lethality test (BSLT) assay, where the results have been presented in Table 4. Mortality percentages of 80% and 90% were achieved as soon as the extract concentration increased to 50 and 1000 mg/L, respectively. Using linear regression, we obtained LC_50_ = 39.12 mg/L.

### 2.7. Molecular Docking Results

**Figure 3:**
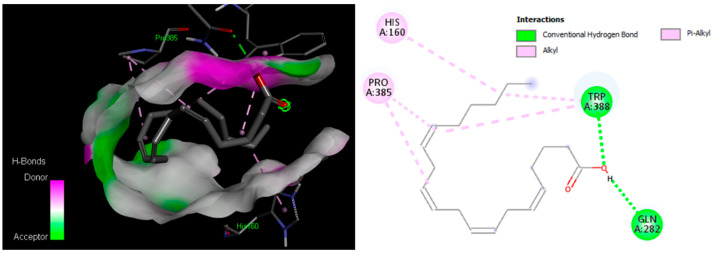
3D (**left**) and 2D (**right**) conformations of ligand–protein complex interaction between arachidonic acid and GLUT9.

The results of molecular docking against the human GLUT9 (hSLC2A9), where the chemical constituents from *H. atra* act as inhibiting ligands, have been presented in Table 5. Redocking with the native ligand revealed their binding affinity of −8.6 kcal/mol, comprised of two hydrogen bonds (Gln283 and Asn288) and seven hydrophobic bonds (He287, Phe379, Glu380, Gly384, Trp388, Phe398, and Gln282). Unfortunately, the scores yielded for the chemical constituents of *H. atra* were lower than that of native ligands. Moreover, the docking scores only ranged from −4.2 to −6.1 kcal/mol with Bis(2-ethylhexyl) phthalate yielded the highest score. As for heptacosanoic acid, methyl ester; arachidonic acid; and (6*Z*,9*Z*,12*Z*,15*Z*)-methyl octadeca-6,9,12,15-tetraenoate, the binding energy reached −6.0 kcal/mol. Using the cut-off of −5.0 kcal/mol for possible interactions between the ligands and protein, we obtained 12 compounds that contribute to the possible inhibition of GLUT9. The 3D and 2D conformation images of the interaction yielded from the molecular docking simulation between GLUT9 and arachidonic acid (the highest in abundance) have been presented in Figure 3.

## 3. Discussion

Extraction using n-hexane solvent in this present study yielded a fatty acids-rich product deriving from *H. atra*. Polyunsaturated (arachidonic acid) and monounsaturated fatty acids (oleic acid) were detected in the extract. This is in agreement with the study investigating the fatty acids profile in *H. atra* collected from a neighboring country—Malaysia that polyunsaturated fatty acids in the sea cucumber are predominated by arachidonic acid [29]. *Holothuria scabra* cultured in Bali Province, Indonesia, was reported to consist of 0.47% monounsaturated fatty acids and 0.29% polyunsaturated fatty acids [30]. The content of arachidonic acid and oleic acid was quite pronounced in *H. scabra*, especially the latter [30]. In a study using four different solvents, the fatty acid profiles were likely to be dependent on the type of the solvents [31]. For instance, arachidonic acid was found in all extracts, but oleic acid was only in the phosphate buffer saline extract [31]. The contents of fatty acids in the extract are dependent on habitat because of different food sources which contribute to the biosynthesis of fatty acids [32].

Herein, the extract administration to a rat model with induced hyperuricemia lowered the serum uric acid level and reached statistical significance when the dosage was 150 mg. In previous studies, anti-hyperuricemic plant extracts have been found to contain fatty acids associated with their inhibitory activity of xanthine oxidase [33]. Celery seed extract which was rich in fatty acids content reduced serum uric acid levels and the activity of xanthine oxidase [34]. Seeds oil from *Sonneratia apetala* containing polyunsaturated fatty acids was reported to have the ability to improve hyperuricemic conditions in vivo [35]. Particularly, in the case of sea cucumbers, hydrolysates from *Apostichopus japonicus* and *Acaudina leucoprocta* respectively could promote uric acid secretion and lowering of serum uric acid with additional benefits of regulating pro and anti-inflammatory cytokines [36]. The promotion of uric acid secretion could be associated with the ability of fatty acids in regulating the expression of the urate transporters, which are renal urate re-absorbers—GLUT9a and GLUT9b [35,36].

Our data in this present study suggested the ability of n-hexane extract from *H. atra* in modulating the expressions of GLUT9a and GLUT9b. In general, the expressions of the urate transporters were lower compared to that of the control group. Nonetheless, increasing the extract dosage of more than 50 mg did not always result in lower GLUT9a and GLUT9b expression. This might be attributed to the bioactive contents which were initially too low in concentration to yield any effects, but later became effective once the dosage increased. Even though both of these splice variants, GLUT9a and GLUT9b, have identical roles in urate kinetics, a recent study showed that the former has higher sensitivity against small anions [37]. Nonetheless, it is still unclear how the expressions were modulated in this present study. Lowered GLUT9a expression is associated with higher uric acid clearance, despite the increase in GLUT9b expression. This phenomenon is indicative that GLUT9a might have a more significant role in renal urate reabsorption as compared with GLUT9b, though it has to be further investigated in future research. In previous studies, the reduction of serum uric acid was followed by the downregulation of GLUT9 [35,36]. An in vivo study has concluded that GLUT9 possesses a significant role in renal urate reabsorption, making the molecule as the target for uricosuric therapies [15].

Herein, based on our molecular docking study using GLUT9 as the target protein [38], the chemical constituents of *H. atra* only had binding affinity scores ranged from −4.0 to −6.0 kcal/mol, where the highest score was achieved by eicosanoic acid, methyl ester (present around 1.44% in the extract). This compound along with arachidonic acid; 11-octadecenoic acid, methyl ester; nonadecanoic acid had a docking score below −5.0 kcal/mol indicative of possible interaction with the target protein. However, the docking scores were found unable to compete with that of the native ligand (docking score = −8.6 kcal/mol). Taken altogether, it is less likely that the chemical constituents of *H. atra* reduce the serum uric acid through GLUT9 activity inhibition via competitive binding [39]. The reduction of serum uric acid by *H. atra* extract is likely to be contributed by the downregulation of GLUT9 expression.

In this present study, ALT and AST were reduced following the treatment using *H. atra* extract. The hepatoprotection and normalizing effect of the extract from *H. atra* against ALT and AST have been witnessed in a published report [40]. Amelioration of the liver could be ascribed to the antioxidant and anti-inflammatory of *H. atra* extract as suggested previously [41,42]. However, in the case of BUN herein, we obtained a significant decrease at an extract dosage of 50 mg, yet the level increased with almost statistical significance at 150 mg. Cytotoxicity assay using *A. salina* supports the fact that the n-hexane extract from *H. atra* is highly cytotoxic. This toxicity could be ascribed to arachidonic acid content in the extract. Endogenous arachidonic acid is a functional component of the cell membrane, where in hyperuricemic conditions it could transform into various inflammatory mediators [43]. Moreover, a deleterious compound, bis(2-ethylhexyl) phthalate, was found in this present study. Bis(2-ethylhexyl) phthalate is a common plasticizer used in polyvinyl chloride manufacturing, in which its contamination and deleterious effects on human health have been notified in several studies [44,45]. Nonetheless, in living organisms (particularly in plants and microbes), this compound is biosynthesized endogenously as a survival mechanism [46].

## 4. Materials and Methods

### 4.1. Materials

The ethanol 96%, dimethyl sulfoxide (DMSO), potassium oxonate, and carboxyl methyl cellulose (CMC) were analytical grade and obtained from Sigma-Aldrich (Selangor, Malaysia). As for ketamine, xylazine, NaCl 0.9%, and allopurinol, they were pharmaceutical grade and purchased from Kalbe Farma (Jakarta, Indonesia). Otherwise stated, all chemicals were used as obtained from the manufacturer without pre-treatment. The solvent ethanol was re-distilled before being used.

Sea cucumber specimen was collected from Simeuleu Islands, Aceh Province, Indonesia in December 2020. The specimen identification based on its morphology and anatomy was carried out in the Marine Biology Laboratory, Faculty of Marine and Fisheries, Universitas Syiah Kuala on 15 December 2020, with voucher no. 003/UN11.1.10/TU/2020. The specimen was confirmed to be *Holothuria atra*.

### 4.2. Extraction of H. atra

*H. atra* specimens were anesthetized using dry ice, sliced open, and the internal organs were removed. The body wall and internal organs were rinsed separately with continuously flowing distilled water and oven-dried at 40 °C before being crushed into powdered simplicia. Dried simplicia derived from the body wall and internal organs were mixed as one sample. Thereafter, the maceration (3 × 24 h) was carried out using n-hexane solvent (1:1). The filtrate was concentrated with a rotary evaporator (40 °C; 30 rpm). The compounds comprised in the extract were analyzed on chromatography–mass spectrometry (GC–MS—QP2020 NX, Shimadzu, Kyoto, Japan), following the suggestion from the previous report [47,48].

### 4.3. Hyperuricemic Animal Model and Treatment

The research protocol has been priorly approved by the ethics committee of the Faculty of Veterinary Medicine, Universitas Syiah Kuala (No. 82/KEPH/XII/2020) Male Wistar rat (*Rattus norvegicus*; *n* = 30) aged 12–14 weeks and weighing 200–300 g were procured from Animal Model Laboratory, Biomedical Research Center, Research Hub-Indonesia. The reason for choosing Wistar rats includes the fact that the reabsorption of serum uric acid resembles what occurs in humans [49]. Moreover, transporters involved in this process have their active sites, such as Val253Ile, conserved in both human and Wistar rats [50]. Firstly, the rats were acclimated at room temperature (22 ± 2 °C) for 7 days through 12 h light–dark cycles and fed with standard feed containing 17% protein ad libitum. Thereafter, the rats made fasting for 6 h before any treatment. The animals were then divided into 6 groups (*n* = 5 each) consisting of normal, control, allopurinol, and three extract groups. Except in the normal group, all rats received potassium oxonate (250 mg/kg body weight in NaCl 0.9% suspension) through intraperitoneal injection to induce hyperuricemia. The normal group was injected intraperitoneally with saline 0.9% for placebo control. The allopurinol group was treated with allopurinol (10 mg/kg body weight). *H. atra* extracts at dosages of 50, 100, and 150 mg body weight were given to rats in the groups of 50 mg, 100 mg, and 150 mg, respectively. The therapies were administered QD (once a day) orally through a nasogastric tube in CMC 0.5% suspension (10 mL) for 3 days. Finally, the rats were sacrificed under ketamine (100 mg/kg) and xylazine (20 mg/kg) following 1-h post-intervention. Rapid body weight reduction (≥200 g) and severe diseases or injuries obtained during the research timeframe were set as humane endpoints.

### 4.4. Determination of Serum Parameters

Abdominal aortic blood was drawn from the animal model, centrifuged (3000 rpm; 10 min), and stored at −20 °C until further use. Analyses of parameters BUN and serum uric acid, creatinine, AST, and ALT were carried out on enzyme-linked immunosorbent assay (ELISA). The serum parameters were determined before (1 h after potassium oxonate injection) and after treatment.

### 4.5. Determination of Gene Expressions of GLUT9a and GLUT9b

Gene expressions of renal GLUT9a and GLUT9b were based on the suggestion of a previously reported study [51]. RNA total was extracted from the renal tissue using TRIzol©, and further extracted to obtain the cDNA using ReverTra AceTM qPCR RT Master Mix with gDNA Remover (TOYOBO) cDNA synthesis kit. The cDNA extraction followed the instructions from the manufacturer. DNA primers used for the amplifications of mGLUT9a and mGLUT9b have been presented (Table 6). Polymerase chain reaction (PCR) was performed with the following cycles: pre-denaturation at 95 °C for 1 min, 40 cycles at 95 °C for 3 s, and followed by another cycle at 60 °C for 20 s. The number of mRNAs was calculated based on the cycle threshold (CT) on Applied Biosystem 7500 v.2.0.6 (Thermo Fisher Scientific, Selangor, Malaysia). Relative mRNA expression was determined with internal normalization with β-actin.

### 4.6. BSLT Assay

n-Hexane extract of *H. atra* was dissolved in saline water in the presence of DMSO 5% (1–3 drops) until the extract concentrations ranged from 25 to 1000 mg/mL. Each extract in various concentrations was added to a vial bottle containing 10 newly hatched *Arthemia salina* L. larvae and then incubated for 24 h. One bottle was only added with saline water mixed with 3 drops of DMSO as the control. This protocol was performed in triplicate. Immobile larvae were considered dead, counted, and compared with those in control to calculate the LC_50_ value.

### 4.7. Docking Simulations

Protein preparation was carried out with Pymol to remove water molecules and ligands attached to the protein. Minimizing energy in the ligands is carried out with an open babel integrated into the PyRx program. The 3D structure of the ligand was obtained from the PubChem database and the 3D image of human GLUT9 (hSLC2A9; PDB ID: 4PYP). The docking process was carried out using Autodock integrated with PyRx version 0.9.5 with Lamarckian Genetic Algorithm parameters. Next, the docking grid was directed to the Quercetin control binding site at AutoGrid Dimensions center X: xx, Y: yy, and Z: zz, and Number of points X: xx, Y: yy, and Z: zz, with a spacing of 0.375 Å. The grid selection was based on the key residues of hSLC2A9, as suggested by a previous study [38]. The interaction visualization was performed on Discovery Studio 2021 and PyMol V.2.5.1 software.

### 4.8. Statistical Analysis

Statistical analysis was carried out on GraphPad Prism version 9.0.0 (GraphPad Software, LLC—San Diego, CA, USA). Determination of the data distribution was based on the Shapiro–Wilk test at α = 0.05. Statistical significance was determined based on paired *t* test and Wilcoxon test for normally and non-normally distributed data, respectively.

## 5. Conclusions

n-Hexane extract from *H. atra*, which is rich with fatty acid contents, has been evidenced to possess anti-hyperuricemic properties in vivo. Moreover, the extract could regulate the urate kinetics via GLUT9a and GLUT9b through unclear mechanisms. Molecular docking simulation suggested the unlikelihood of competitive inhibition as the mechanism. Serum ASR and ALT levels were reduced in rats treated with *H. atra* extract. In the future, it is worth investigating the hepatoprotective activity of the extract by observing the changes in liver functions as well as histopathological images of the liver. It is worth mentioning that our findings on the increase in BUN level and highly cytotoxic activity of the extract alarm its usage and urge further investigation regarding the safe dosage range. Further studies to emphasize the different renal urate reabsorption by the two splice variants of GLUT9 for uricosuric drugs’ targets are warranted.

## Figures and Tables

**Table 1:** GC–MS profile of n-hexane from *H. atra*.

No.	Compound	Retention Time (min)	Area (%)
1	2-Pentadecyn-1-ol	16.488	5.99
2	1-Octyn-3-ol, 4-ethyl-	16.538	2.57
3	1-Dodecene	17.571	1.49
4	Cyclopropanepentanoic acid, 2-undecyl-, methyl ester, trans	18.821	1.49
5	Pentadecanoic acid, 14-methyl-, methyl ester	19.031	6.62
6	Oleic acid	19.267	1.51
7	Nonadecanoic acid	19.452	2.40
8	1-Tetradecene	19.754	1.47
9	Heptacosanoic acid, methyl ester	21.090	5.38
10	1-Tetradecene	21.747	1.52
11	Arachidonic acid	22.394	44.61
12	(6*Z*,9*Z*,12*Z*,15*Z*)-Methyl octadeca-6,9,12,15-tetraenoate	22.455	6.35
13	Cyclopropanepentanoic acid, 2-undecyl-, methyl ester, trans	22.740	4.16
14	9,12,15-Octadecatrienal	22.840	2.88
15	Eicosanoic acid, methyl ester	22.977	1.44
16	Oleic acid	23.153	4.37
17	1-Hexacosanol	23.573	1.28
18	Bis(2-ethylhexyl) phthalate	24.801	2.20
19	11-Octadecenoic acid, methyl ester	25.352	1.05
20	11-Octadecenoic acid, methyl ester	26.188	1.21

**Table 2:** Levels of serum AST and ALT before and after the treatment.

Parameters	Before	After	*p*-Value
AST, Mean ± SD (IU/L)			
Normal	139.2 ± 11.43	140.4 ± 2.074	0.8333
Control	208.4 ± 49.13	206.4 ± 55.13	0.7419
Allopurinol	224.4 ± 47.68	151.2 ± 22.07	0.0198 *
50 mg	214.8 ± 55.74	145.4 ± 15.87	0.0237
100 mg	216.0 ± 44.96	148.8 ± 20.07	0.0070 **
150 mg	224.4 ± 29.64	132.2 ± 32.88	0.0010 **
ALT, Mean ± SD (IU/L)			
Normal ^a^	80.00 ± 7.906	80.8 ± 5.848	0.7205
Control ^a^	136.2 ± 41.57	133.8 ± 36.95	0.8750
Allopurinol ^a^	138.6 ± 82.86	95.6 ± 11.48	0.1875
50 mg	101.4 ± 12.76	94.8 ± 8.871	0.4353
100 mg	124.4 ± 46.32	98.2 ± 21.81	0.0830
150 mg	112.0 ± 14.49	78.2 ± 8.64	0.0302 *

Normal ranges for serum AST and ALT in Wistar rats are 50–150 IU/L and 10–40 IU/L, respectively [27]. ^a^ Otherwise stated, the analysis was carried out using paired *t*-test. * Statistically significant at *p* < 0.05 and ** very significant at *p* < 0.01. AST: Aspartate aminotransferase; ALT: Alanine aminotransferase.

**Table 3:** Levels of BUN and serum creatinine before and after the treatment.

Parameters ^a^	Before	After	*p*-Value
BUN, Mean ± SD (mg/dL)			
Normal	23.00 ± 9.354	22.80 ± 8.468	0.7040
Control	19.80 ± 5.263	20.60 ± 7.162	0.7003
Allopurinol	22.80 ± 10.38	19.40 ± 4.037	0.4492
50 mg	21.80 ± 13.22	17.40 ± 5.814	0.0222 *
100 mg	13.40 ± 5.60	18.40 ± 8.562	0.5392
150 mg	14.80 ± 5.36	18.80 ± 8.167	0.0577
Creatinine, Mean ± SD (mg/dL)			
Normal ^b^	0.66 ± 0.134	0.68 ± 0.192	>0.9999
Control	0.70 ± 0.235	0.62 ± 0.109	0.5122
Allopurinol ^b^	0.68 ± 0.192	0.64 ± 0.089	0.7500
50 mg	0.62 ± 0.217	0.54 ± 0.182	0.5769
100 mg ^b^	0.66 ± 0.114	0.62 ± 0.447	0.6250
150 mg	0.60 ± 0.192	0.68 ± 0.0837	0.2420

Normal ranges for serum BUN and creatinine are 15–22 mg/dL and 0.4–0.8 mg/dL, respectively [28]. ^a^ Otherwise stated, the analysis was carried out using paired *t*-test. ^b^ Analyzed using the Wilcoxon test. * Statistically significant at *p* < 0.05. Wilcoxon test. BUN: Blood urea nitrogen.

**Table 4:** Results from BSLT assay of the n-hexane extract from *H. atra* using *Artemia salina*.

Concentration (mg/L)	Dead, Mean ± SD	Mortality (%)	Probit
10	3.33 ± 2.08	33.33	4.56
25	4.67 ± 4.73	46.67	4.92
50	8.00 ± 2.00	80.00	5.84
75	5.33 ± 0.58	53.33	5.08
100	2.67 ± 2.89	26.67	4.39
250	7.00 ± 1.00	70.00	5.52
500	8.33 ± 2.08	83.33	5.95
750	7.33 ± 2.31	73.33	5.61
1000	9.00 ± 0.00	90.00	6.28
Linear regression equation	y = 0.682x + 3.914		
LC_50_ (mg/L)	39.12		

Total *n* = 10.

**Table 5:** Molecular docking results targeting GLUT9.

Compounds	Binding Energy (kcal/mol)	Hydrogen Bond	Hydrophobic Bond
2-Pentadecyn-1-ol	−4.8	Gln282, Trp 388	Ile164, Phe26
1-Octyn-3-ol, 4-ethyl-	−4.2	Gln282	Ile164, Glu 380
1-Dodecene	−4.3		Pro385, Phe379, Ile164, Val165, Trp388
Pentadecanoic acid, 14-methyl-, methyl ester	−5.1	Trp388, Gln282, Asn411	Phe378, Ile287, Ile164, Phe26
Nonadecanoic acid	−5.5	Asn411, Trp388	Ile164, Pro385, Val165
1-Tetradecene	−4.3		Val165, Ile164, Pro385, Trp388
Heptacosanoic acid, methyl ester	−6.0		Trp412, Trp388, Phe26, Ile164
Arachidonic acid	−6.0	Trp388, Gln282	His160, Pro385
(6*Z*,9*Z*,12*Z*,15*Z*)-Methyl octadeca-6,9,12,15-tetraenoate	−6.0	Asn411, Trp388, Gln282, Gln283	Pro385, Val165, Ile164, His160
Cyclopropanepentanoic acid, 2-undecyl-, methyl ester, trans	−5.7	Asn411, Trp388	His160 Ile164 Val165 Pro385
9,12,15-Octadecatrienal	−5.1		Pro385 Ile164 His160 Trp388 Val165 Phe26
Eicosanoic acid, methyl ester	−5.4	Trp388, Asn411	Ile164, Phe291, Val165, Pro385, Phe379
Oleic acid	−5.5	Asn411, Gln282, Trp388	Phe26, Pro385, Ile164, Val165
1-Hexacosanol	−5.6	Ser80	Trp412, Trp388, Phe26, Ile164, Ile287
Bis(2-ethylhexyl) phthalate	−6.1	Gln282, Asn411	Pro385, Trp388, Ile164, His 160
11-Octadecenoic acid, methyl ester	−5.3	Gln282, Gln283	Glu380, Ile164, Phe26, Pro385
Native ligand	−8.6	Gln283, Gln282, Asn288, Glu380	Pro385, Trp388, Phe398
Allopurinol	−4.9	Asn317, Glu380 Asn288	Ile168

**Table 6:** Primers used to determined the expressions of GLUT9a and GLUT9b using qualitative PCR.

Molecule	Sequence
β-actin	F: 5′-CCTAAGGCCAACCGTGAAAAGATG-3′
	R: 5′-GTCCCGGCCAGCCAGGTCCAG-3′
GLUT9a	F: 5′-GGGTCACCAGCAGAGGAG-3′
	R: 5′-TGGACCAAGGCAGGGACAA-3′
GLUT9b	F: 5′-AACTCCGCAGAAACCAAGGAAAGC-3′
	R: 5′-TTCAAAGAGAAGGTAGCGTGGGCT-3′

F: Forward; R: Reverse.

## Data Availability

The data presented in this study are available on request from the first author. The data are not publicly available due to the fact that this study is still ongoing.
